# Functional states of rat cortical circuits during the unpredictable availability of a reward-related cue

**DOI:** 10.1038/srep37650

**Published:** 2016-11-21

**Authors:** Iván Fernández-Lamo, Raudel Sánchez-Campusano, Agnès Gruart, José M. Delgado-García M

**Affiliations:** 1 Division of Neurosciences, Pablo de Olavide University, Seville-41013, Spain

## Abstract

Proper performance of acquired abilities can be disturbed by the unexpected occurrence of external changes. Rats trained with an operant conditioning task (to press a lever in order to obtain a food pellet) using a fixed-ratio (1:1) schedule were subsequently placed in a Skinner box in which the lever could be removed randomly. Field postsynaptic potentials (fPSPs) were chronically evoked in perforant pathway-hippocampal CA1 (PP-CA1), CA1-subiculum (CA1-SUB), CA1-medial prefrontal cortex (CA1-mPFC), mPFC-nucleus accumbens (mPFC-NAc), and mPFC-basolateral amygdala (mPFC-BLA) synapses during lever IN and lever OUT situations. While lever presses were accompanied by a significant increase in fPSP slopes at the five synapses, the unpredictable absence of the lever were accompanied by decreased fPSP slopes in all, except PP-CA1 synapses. Spectral analysis of local field potentials (LFPs) recorded when the animal approached the corresponding area in the lever OUT situation presented lower spectral powers than during lever IN occasions for all recording sites, apart from CA1. Thus, the unpredictable availability of a reward-related cue modified the activity of cortical and subcortical areas related with the acquisition of operant learning tasks, suggesting an immediate functional reorganization of these neural circuits to address the changed situation and to modify ongoing behaviors accordingly.

Cognitive flexibility allows us to respond to changing environmental events (e.g., the presence of new context or cues) leading to the generation of adaptive behaviors^1^. These adaptations require the modification of previously acquired stimulus-response associations^2^,^3^,^4^. The detection of novel conditions must take place before the adapted behavior occurs, since several psychological factors, like reward^5^, attention^6^ and novelty^7^, can modify ongoing cerebral functions^8^. Accordingly, it is important to understand brain processes by which changes in the availability of a reward-related cue are compared with the functional states corresponding to previously acquired motor situations and how this detection will modify subsequent behaviors.

Neural oscillations emerge from the network of excitatory and inhibitory synaptic connections specific of each neural center and result from the phase synchrony of cell assemblies^9^. Neural oscillations are thought to contribute to the dynamic coupling in neural communication between related brain areas underlying different cognitive processes^10^,^11^,^12^,^13^,^14^. One of the most prominent and best studied oscillations is the theta (3–12 Hz) rhythm, which has been shown to play a role in different learning and memory functions^12^,^15^,^16^,^17^,^18^. Information flow through theta oscillations between the hippocampus and the neocortex points to this network as a core component in cognitive processes and subsequent behaviors^15^,^19^,^20^. While the hippocampus has been proposed to create context representation in space and time^21^,^22^,^23^ and to facilitate the formation of memory traces important to long-term memory storage^24^, cortical structures such as mPFC have a special role in cognitive processes involved in the selection, timing, and execution of particular behaviors^25^,^26^,^27^,^28^,^29^,^30^. Finally, subcortical structures as the NAc and the BLA have been related to different motivational and rewarding learning tasks^16^,^31^,^32^,^33^.

Within the hippocampus, the CA1 region has been related to appetitive and consummatory behaviors during operant conditioning tasks^34^, as well as during novelty detection^35^,^36^. The SUB is a hippocampal output structure receiving synaptic excitation from CA1 pyramidal cells, and represents an important connecting node for processing spatial information and body movements. In particular, the dorsal SUB is necessary for the memory of self-motion cues^37^ and object recognition^38^, as well as being involved in hippocampal-ventral tegmental area loops for rewarding long-term memories^7^,^33^.

Changes in synaptic weights during the acquisition of classical^24^ and instrumental^34^ conditioning paradigms have been reported for the above-mentioned neural centers, as well as during the performance of inhibitory avoidance learning^39^ and object recognition^40^,^41^ tasks. In addition, brain state can change rapidly such as in the case of attention, and transiently modulate neural responsiveness^42^,^43^,^44^,^45^,^46^. In the latter case, it is reasonable to think that the relative synaptic strength of memory-related synapses will be modified when previously learned behaviors have to be readapted in response to new and unpredictable environmental constrains.

Our hypothesis is that neural information (both stored and sensorial) coded and transmitted between the above mentioned brain areas is continuously modified by cognitive states. These ongoing functional states may be affected by changes in synaptic strength^21^,^24^ and/or in the spectral powers of their respective ongoing oscillations^12^. It is expected that these neural activities are modified by any disturbance presented during the performance of already acquired tasks and, therefore, they can be recorded as differences in synaptic strength and spectral patterns, before new adaptive motor responses occur. Present results collected from alert behaving rats presented with an unpredictable situation seem to support these contentions.

## Results

### Instrumental conditioning of behaving rats with a fixed-ratio (1:1) schedule

**Figure 1:**
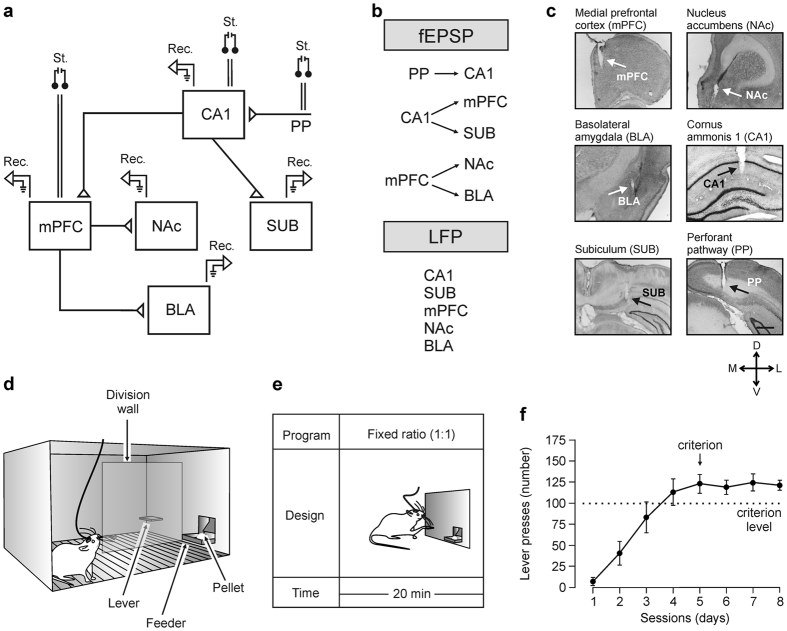
Experimental design. (**a,b**) Rats were divided in four groups and implanted with: (i) bipolar stimulating (St.) electrodes in the left PP and recording (Rec.) electrodes in the ipsilateral hippocampal CA1 area; (ii) stimulating electrodes in the left CA1 area and recording electrodes in the ipsilateral mPFC and SUB; (iii) stimulating electrodes in the mPFC and recording electrodes in the ipsilateral NAc and the BLA; and (iv) recording electrodes in the left CA1, SUB, mPFC, NAc, and BLA. (**c**) Representative photomicrographs of recording electrodes implanted in the mPFC, NAc, BLA, CA1, and SUB and of stimulating electrodes implanted in the PP. Calibration bar is 0.5 mm. Abbreviations: D, L, M, and V, dorsal, lateral, medial, and ventral. (**d,e**) In a first experimental step, animals were trained in a Skinner box to press a lever to obtain a pellet of food with a fixed-ratio (1:1) schedule. Sessions lasted for 20 min. (**f**) Acquisition curve collected from a group of animals (group 1, PP-CA1, n = 10) during the fixed-ratio (1:1) task. Each animal was switched to the following experimental task on reaching asymptotic values for ≥3 consecutive sessions (arrow).

As detailed in Methods, experimental rats were prepared for the chronic recording of fPSPs at PP-CA1, CA1-SUB, CA1-mPFC, mPFC-NAc, and mPFC-BLA synapses (Fig. 1a,b) or of LFPs at CA1, SUB, mPFC, NAc, and BLA sites (Fig. 1a–c)^21^,^24^,^27^.

In a preliminary series of experiments, rats were trained for the acquisition of an instrumental conditioning with a fixed-ratio (1:1) schedule—i.e., each lever press was reinforced with a food pellet (Fig. 1d,e). Training sessions were performed daily and lasted for 20 min. Animals progressively improved their performance in the Skinner box with the successive sessions (Fig. 1f). The criterion for proper acquisition was to press the lever a minimum of 100 times per session for two successive sessions (Fig. 1f). Acquired data were best fitted with sigmoid curves (*r* ≥ 0.97; *P* < 0.001; not illustrated). For the sake of homogeneity, animals that did not acquire the task during the first five sessions (a total of 4 animals; ≤2 per group) were rejected from the study and substituted by other ones. No fPSPs or LFPs were recorded during these training sessions.

**Figure 2:**
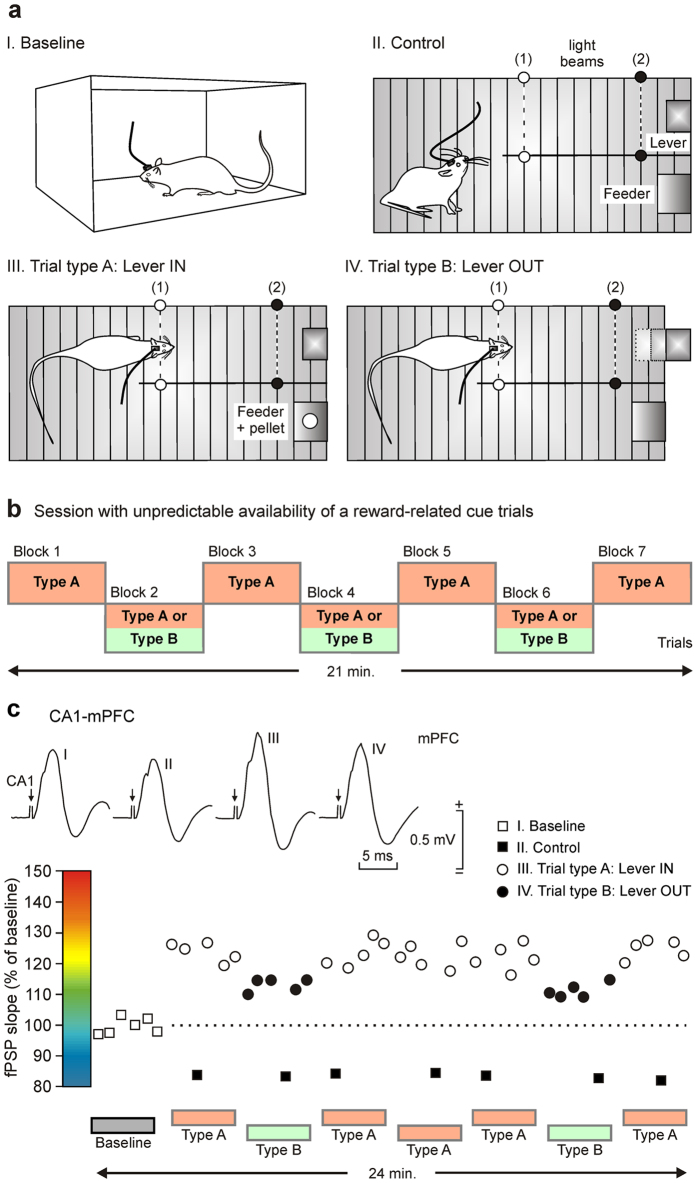
Experimental design and evolution of fPSPs recorded during the unpredictable task. (**a**) For the unpredictable task, animals were placed in a modified Skinner box provided with two light beams located at 10 cm (1) and 2 cm (2) from the lever. fPSPs were evoked and recorded in four different situations: (i) I. Baseline: with the animal moving around in an auxiliary Perspex box located beside the Skinner box used for the experiments; (ii) II. Control: with the animal resting outside the lever and/or feeder areas; (iii) III. Trial type A, Lever IN: the lever was always available when the animal approached it across the strip with the two (1, 2) light beams; and (iv) IV. Trial type B, Lever OUT: the lever was removed when the animal’s head cut the 1st light beam. In the last two cases, electrical stimulation of the selected synapses was carried out when the animal’s head crossed the 2nd light beam (black dots). (**b**) The single unpredictable session lasted for 21 min and included seven periods of 3 min. In the odd blocks (1, 3, 5 and 7), the lever was always available (Type A trials), whilst in the even blocks (2, 4 and 6), the lever was available or removed at random (Type A or Type B trials). Inside each block, all the trials were equal (either type A or type B). (**c**) Evolution of fPSPs evoked at the CA1-mPFC synapse in a representative rat across a complete unpredictable session. fPSPs were evoked with intervals ≥ 20 s. Note the different fPSP amplitudes evoked during the four experimental situations: (i) Baseline; (ii) Control; (iii) Trial A, Lever IN; and (iv) Trial B, Lever OUT.

**Figure 3:**
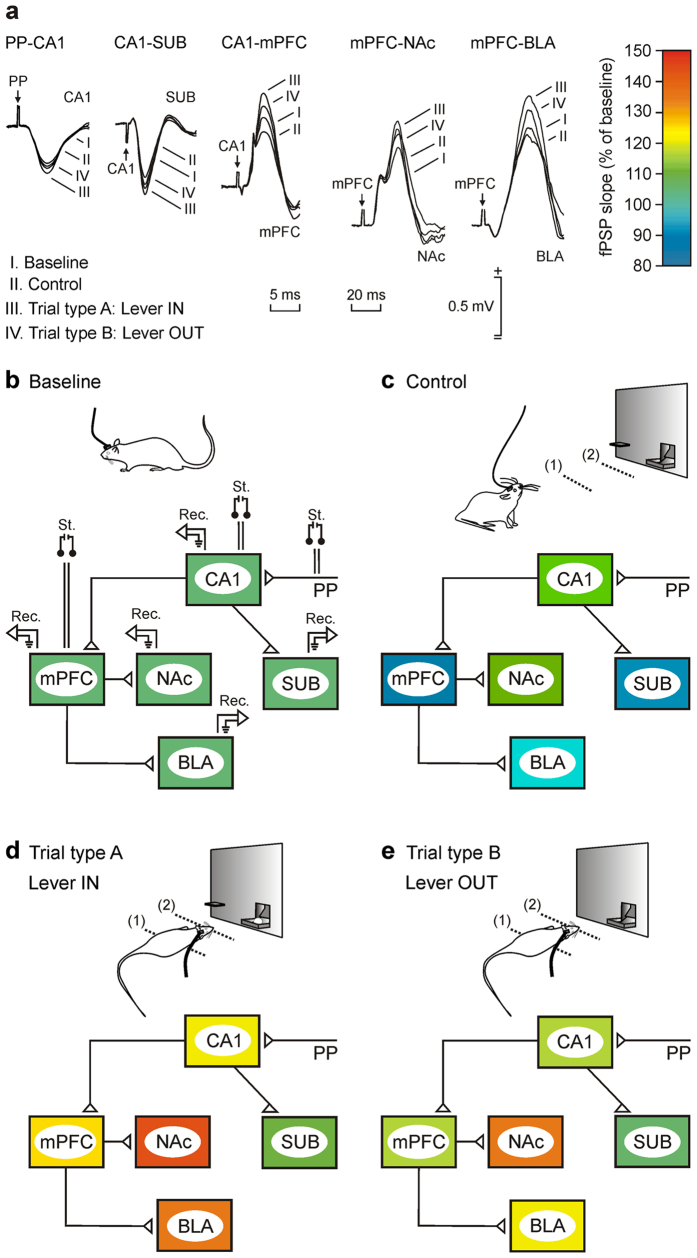
Comparative analysis of fPSPs recorded at the five selected synapses during the unpredictable task. (**a**) fPSPs (averaged five times) recorded from the five selected synapses in the baseline box, during control resting behavior, and during situations Type A and Type B. Time calibration is 5 ms is for the three left set of records and 20 ms for the two right set of records. Amplitude calibration is for all records. (**b–e**) A color representation of the whole synaptic network included in this study with indication of the stimulating and recording points. The color code indicating activity-dependent changes in synaptic strength is illustrated in (**a**). (**b**) Baseline values for fPSPs evoked at these five synapses were collected with the animals located in a Perspex (baseline; see Fig. 2a) box and were adjusted to a 100% value. (**c**) Note that most fPSPs decreased in amplitude in the control situation (with the animal resting in the Skinner box far away from lever and feeder areas). (**d,e**) The left diagram (**d**) illustrates changes in synaptic strength that took place for Type A trials (lever always in), whilst the right diagram (**e**) illustrates changes in synaptic strength that took place for Type B trials (lever always out).

After reaching criterion, rats were trained with the fixed-ratio (1:1) schedule for three additional sessions (Fig. 1f). During these last three training sessions, individual rats were recorded in the auxiliary box selected for baseline recordings (Fig. 2a). Each rat was stimulated at increasing intensities to determine the input/output profiles for each recording site. Stimulus intensities were selected at about 30–40% of the intensity necessary for evoking a maximum response (Figs 2c and 3a).

### Changes evoked in fPSPs recorded at the five selected synapses during performance of the unpredictable task

The unpredictable task was carried out in a modified Skinner box provided with a division wall (Fig. 2a) located between lever and feeder modules and with two light beams (1 and 2) placed at 10 cm and 2 cm, respectively, from the lever. During type A (Lever IN) trials, the lever was available as usual, but during type B (Lever OUT) trials the lever was removed when the animal crossed the light beam (1). As illustrated in Fig. 2b, the session with the unpredictable availability of reward-related cue trials was divided in 3-minute blocks during which the lever could be IN (Type A trials) or it could be presented either IN or OUT at random (Type A or Type B trials; Fig. 2b).

Each animal received just one session (21 min) with unpredictable trials in which it was stimulated at the implanted site whilst fPSPs were recorded at the selected recording sites. Even in the transient absence of the lever, the rats’ performance during the unpredictable task presented high values (≥76 pellets/session, n = 30 rats).

fPSPs were evoked in four different behavioral situations (Figs 2a,b and 3a). In order to avoid neural activity interferences with preceding stimuli, the electrical pulses were presented with intervals >20 s. Importantly, fPSPs evoked the first time that the animal approached in the Lever OUT situation were discarded from further analysis, because the uncertainty of the presence or absence of the lever could be considered only during subsequent approaches to the lever site.

Changes taking place in fPSPs evoked in a selected animal in the CA1-mPFC synapse across a complete session with unpredictable availability of the lever are shown in Fig. 2c. As can be seen, fPSP slopes (compared with baseline recordings; white squares) increased during trials in which the lever was IN (white circles) and, interestingly, decreased during trials in which the lever was OUT (black circles), and presented minimum values when the animal was resting in the training cage (control recordings, black squares). These results suggested that the strength of the synaptic activity at the CA1-mPFC was rapidly modified depending on the behavioral situation. No decreasing or increasing trends in fPSP slopes were observed across the whole session. fPSPs evoked at the five selected synapses presented short, stable latencies indicative of their probable monosynaptic nature (Fig. 3a), but their amplitude and slope were modified for the different behavioral situations included in the present study.

Figures 2c and 3b–e illustrate the changes in slope of fPSPs evoked at the different synapses. Taking as 100% fPSP slopes evoked during baseline recordings (see color code in Fig. 3a), it can be seen that fPSPs evoked at the different synapses (n = 10 animals and ≥20 electrodes per synapse) presented different evolutions during the three behavioral situations (i.e., Control, Lever IN, and Lever OUT).

fPSPs evoked at the PP-CA1 synapse increased significantly in slope [One-way RM ANOVA *F*-test; *F*_(3,27,141)_ = 35.655; *P* < 0.001; partial η^2^ = 0.43], in comparison with baseline values, for the three selected behavioral situations (Control, Lever IN, and Lever OUT). Interestingly, no statistically significant differences (Holm-Sidak test; *P* = 0.128) were observed for fPSPs evoked during these three situations, indicating that this synapse was only modified by the different contexts.

fPSPs evoked at the CA1-SUB synapse presented some significant [*F*_(3,27,194)_ = 4.257; *P* < 0.05; partial η^2^ = 0.06] differences between the four recorded behaviors. Firstly, fPSPs evoked at the CA1-SUB synapse decreased significantly (Holm-Sidak test; *P* < 0.001) in slope in the control situation as compared with baseline values and presented a slight, but significant (*P* = 0.012), increase for Lever IN but not for Lever OUT (*P* = 0.831) situations. fPSPs evoked for Lever IN were significantly (*P* < 0.05) larger than those evoked for Lever OUT.

Similar findings were observed for the CA1-mPFC [*F*_(3,27,185)_ = 46.965; *P* < 0.001; partial η^2^ = 0.43] and mPFC-BLA [*F*_(3,27,57)_ = 49.174; *P* < 0.001; partial η^2^ = 0.72] synapses—namely, that fPSP slopes decreased significantly (Holm-Sidak test; *P* < 0.001 for the CA1-mPFC synapse, and *P* < 0.01 for the mPFC-BLA synapse) for control situations, whilst they increased for Lever IN (*P* < 0.001 for both synapses) and Lever OUT (*P* < 0.001 for both synapses) situations.

Finally, fPSPs evoked at the mPFC-NAc synapse increased significantly [*F*_(3,27,51)_ = 95.897; *P* < 0.001; partial η^2^ = 0.85] for the three situations (Control, Lever IN, and Lever OUT) as compared with baseline values (Holm-Sidak test; *P* < 0.001 for all comparisons).

It should be noted that, although fPSP slopes increased in all situations with respect to baseline and control values (Fig. 3b–e), increases evoked by the Lever IN situation were significantly (Holm-Sidak test; *P* < 0.05 for all comparisons) larger than those evoked by the Lever OUT situation for all of the synapses included in this study (CA1-SUB, CA1-mPFC, mPFC-NAc, and mPFC-BLA), apart from the PP-CA1 one.

In summary, the slope of fPSPs evoked at the five synapses included in this study increased when the animal approached the lever, as compared with values collected during baseline and control records. Importantly, fPSP slopes decreased for Lever OUT situations in four of these synapses (CA1-SUB, CA1-mPFC, mPFC-NAc, and mPFC-BLA) as compared with values collected during Lever IN situations. A global perspective of the functional states of the five selected synapses for the four different behavioral situations is illustrated in Fig. 3b–e.

### Analysis of LFPs recorded at the five selected cortical and subcortical sites during the performance of the unpredictable task

**Figure 4:**
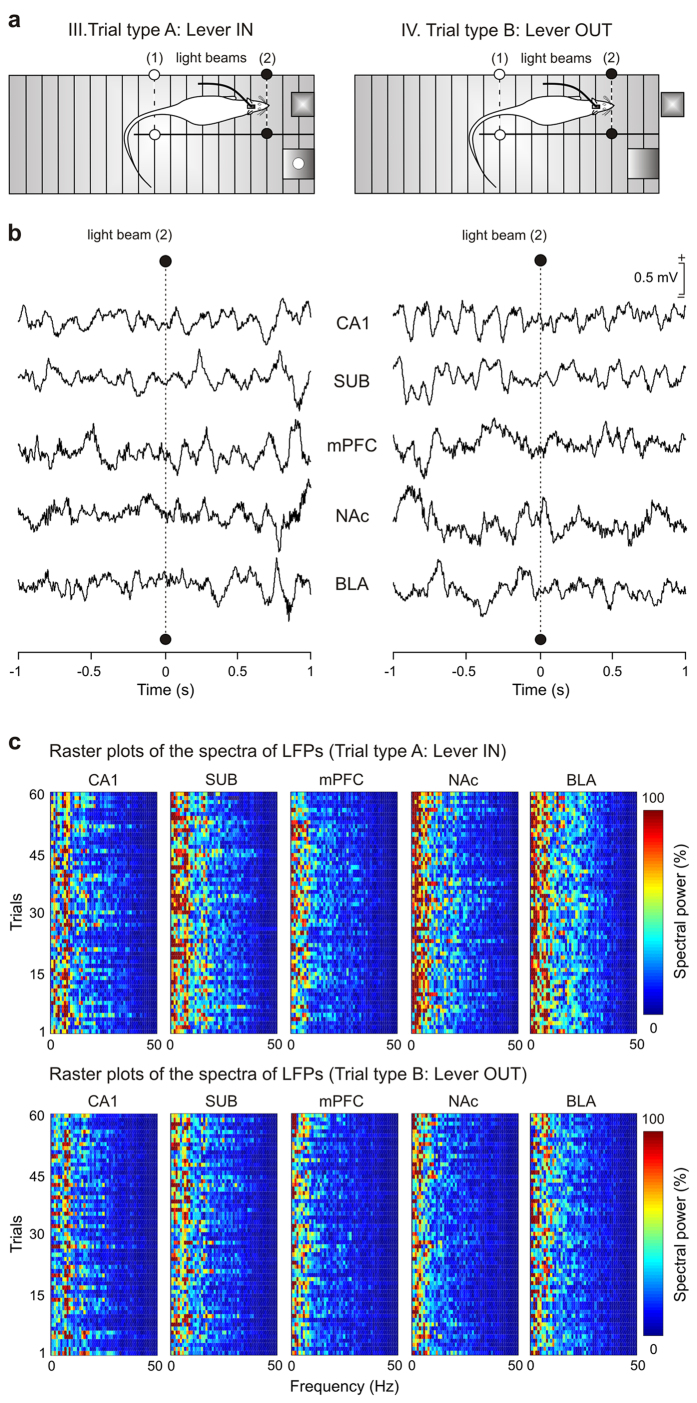
Changes in LFPs evoked at the five recording areas during Type A (Lever IN) and Type B (Lever OUT) trials. (**a**) LFPs were recorded at the five (CA1, SUB, mPFC, NAc, BLA) selected areas from 1 s before to 1 s after the animal’s head cut (dotted line) the 2nd light beam (2, black dots), during both Type A (Lever IN) and Type B (Lever OUT) trials. (**b**) Representative samples (35/electrode) of 2-seconds epochs of LFP activity collected from the five cortical and subcortical areas for situations A and B. (**c**) Color raster displays of the power spectra (spectral power, in %, see the color bar; and frequency, in Hz, see x-axis) across 60 trials (see y-axis) of LFPs recorded from the five different brain sites and for the two experimental conditions (Trial type A: Lever IN; and Trial type B: Lever OUT). LFPs were selected from 1 s before to 1 s after crossing the 2^nd^ light been (2) as illustrated in (**b**). Spectral powers presented predominant values in the frequency range of 1–25 Hz with the low-theta band (3–8 Hz) as the fundamental frequency band. Spectral powers were normalized according to their maximum inter-channel (sites) value.

We were also interested in determining the putative changes in LFP evoked in the five recording sites included in the present study during the same experimental situation. Thus, in a second series of experiments, a new group of animals received the same session containing trials with unpredictability access to the lever that provided a food pellet. During this session, LFPs were recorded in two situations (Trial type A, and Trial type B), but in the absence of any electrical stimulus. Figure 4a illustrates the experimental design and Fig. 4b shows representative recordings collected from CA1, SUB, mPFC, NAc, and BLA from 1 s before to 1 s after the animal crossed the 2nd light beam (black dots). Figure 4c illustrates the raster plots (frequency vs. trials) of power spectra (n = 60) collected from each recording site (time window = 2 s) during type A (Lever IN; Fig. 4c, top set of spectra) and type B (Lever OUT; Fig. 4c, bottom set of spectra) trials. A visual inspection of the raster plots suggested a higher power (in the theta band) for spectra collected during the Lever IN situation for four recording sites (SUB, mPFC, NAc, and BLA), with no noticeable changes between spectra collected from the hippocampal CA1 area during the two experimental (Lever IN and Lever OUT) situations. It can be seen that the spectral powers of LFPs collected during the Lever OUT situation decreased in both sub-bands of theta band (low-theta, 3–8 Hz; high-theta, 8–12 Hz) in comparison to the LFP powers collected form the same time window (2 s) during Lever IN situation, most notably in the raster plots of SUB, mPFC, NAc, and BLA power spectra.

**Figure 5:**
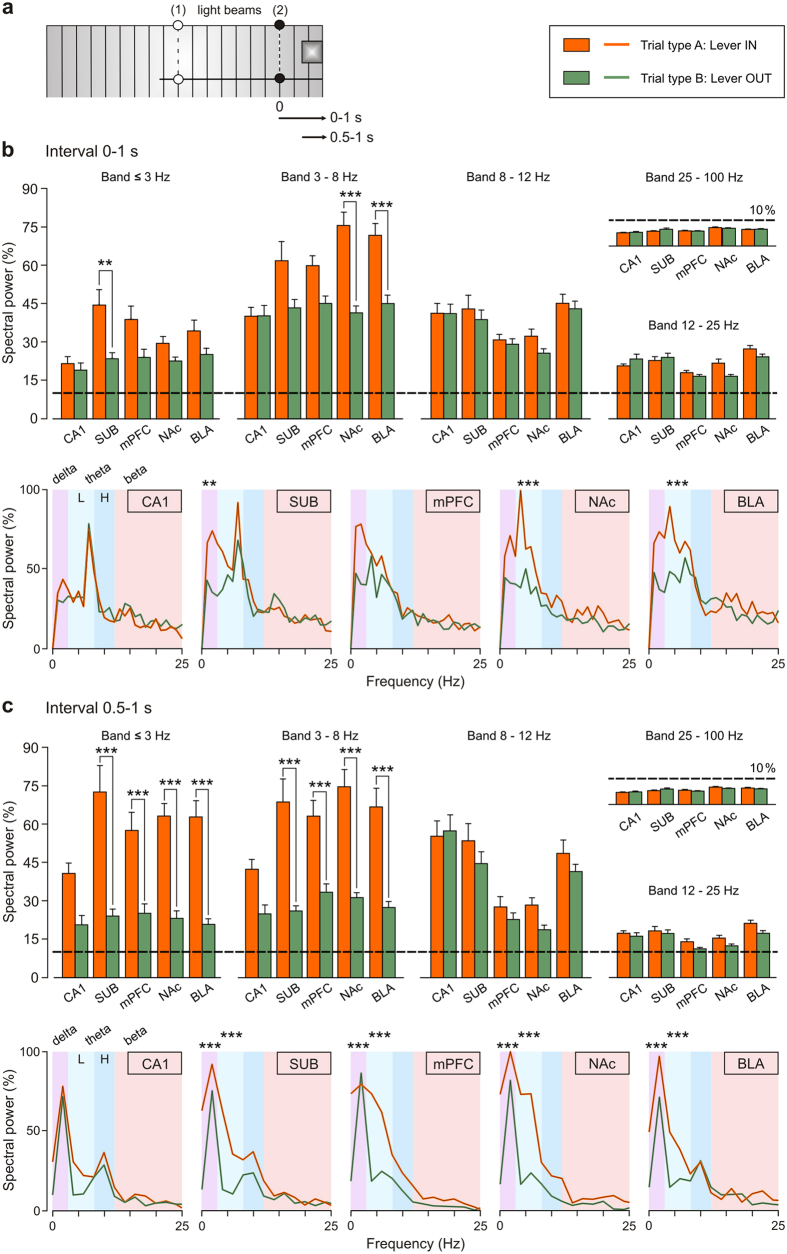
Histograms for the mean spectral powers of LFPs recorded from the five recording areas during Type A (Lever IN) and Type B (Lever OUT) trials. (**a**) The analysis was carried out for LFPs recorded from 0 to 1 s or from 0.5 s to 1 s after the light beam (2) was cut. The color code for type A (Lever IN) and type B (Lever OUT) trials is indicated. (**b**) Spectral analysis for LFPs acquired during the 1st second after the light beam (2) was cut. The maximum power in the delta band [*F*_(1,9,118)_ = 9.98; *P* < 0.01] was presented for LFPs from SUB electrodes during the performance of type A trials (Lever IN, orange bars). In contrast, in the low-theta band, maximum powers were presented for LFPs recorded at the subcortical sites reaching values significantly different at NAc [*F*_(1,9,118)_ = 34.4; *P* < 0.001] and BLA [*F*_(1,9,118)_ = 20.87, *P* < 0.001] from those collected during the performance of the type B trials (Lever OUT, green bars). (**c**) Spectral analysis for LFPs acquired from 0.5 s to 1 s after the light beam (2) was cut. Note that if the time window is reduced to the final 500 ms (i.e., with the rat at the lever site) significant statistical differences (*P* < 0.001) in power between type A (Lever IN) and type B (Lever OUT) trials were obtained for all the recording sites (SUB, mPFC, NAc, and BLA) except for CA1. Mean spectral powers of LFPs in the high-theta, beta, and gamma bands did not present significant differences (*P* > 0.05) at any of the recording sites. At the bottom of both (**b**) and (**c**) are illustrated mean power spectra computed from LFPs recorded at CA1, SUB, mPFC, NAc and BLA areas. The frequency bands [delta, low (L)-theta, high (H)-theta, and beta] are delimited by different color. For the multiple-comparisons with significant differences between mean spectral powers, the significance level is indicated (**P < 0.01; ***P < 0.001). Data are represented by the Mean ± SEM.

A quantitative analysis of spectral powers computed from LFPs recorded during both Lever IN and Lever OUT situations was carried out with the help of the fast Fourier transform (FFT) method (Fig. 5). For this calculation, two specific LFP intervals were taken (Fig. 5a): interval 1, 1st second after the light beam (2) was crossed (Interval 0–1 s); and interval 2, from 0.5–1 second after the light beam (2) was crossed (Interval 0.5–1 s). Spectral analysis carried out for LFPs recorded during Interval 0–1 s indicated that in the delta band (0.1–3 Hz), the maximum value of spectral power [One-way ANOVA *F*-test; *F*_(1,9,118)_ = 9.98; *P* < 0.01; η^2^ = 0.08; Cohen’s d = 0.58; 95% CI: 0.22–0.95] was determined at SUB during the performance of the type A (Lever IN) trials (Fig. 5b). In contrast, in the low-theta band (3–8 Hz), the maximum spectral powers were determined for LFPs recorded at the subcortical sites, reaching values significantly different at NAc [*F*_(1,9,118)_ = 34.4; *P* < 0.001; η^2^ = 0.23; Cohen’s d = 1.08; 95% CI: 0.70–1.46] and BLA [*F*_(1,9,118)_ = 20.87, *P* < 0.001; η^2^ = 0.15; Cohen’s d = 0.84; 95% CI: 0.47–1.21] from those collected during the performance of the type B (Lever OUT) trials (Fig. 5b). However, when the time window was reduced to the final 500 ms (Interval 0.5–1 s: i.e., with the rat at the lever site, Fig. 5c), there were significant statistical differences (Holm-Sidak test; *P* < 0.001 for all comparisons) in the mean spectral powers between Lever IN and Lever OUT conditions for all the recording sites (SUB, mPFC, NAc, and BLA) except for CA1. Note that the spectral powers of the LFPs recorded at CA1 did not reach significant differences (Holm-Sidak test; *P* > 0.05 for all comparisons) between Lever IN and Lever OUT conditions in any frequency band (Fig. 5b,c), or between the five selected bands during trials type A (Lever IN; Fig. 5b,c). Finally, mean spectral powers of LFPs in the high-theta (8–12 Hz), beta (12–25 Hz), and gamma (25–100 Hz) bands did not present significant differences (Holm-Sidak test; *P* > 0.05 for all comparisons) at any of the recording sites when comparing the two conditions (Lever IN vs. Lever OUT).

**Figure 6:**
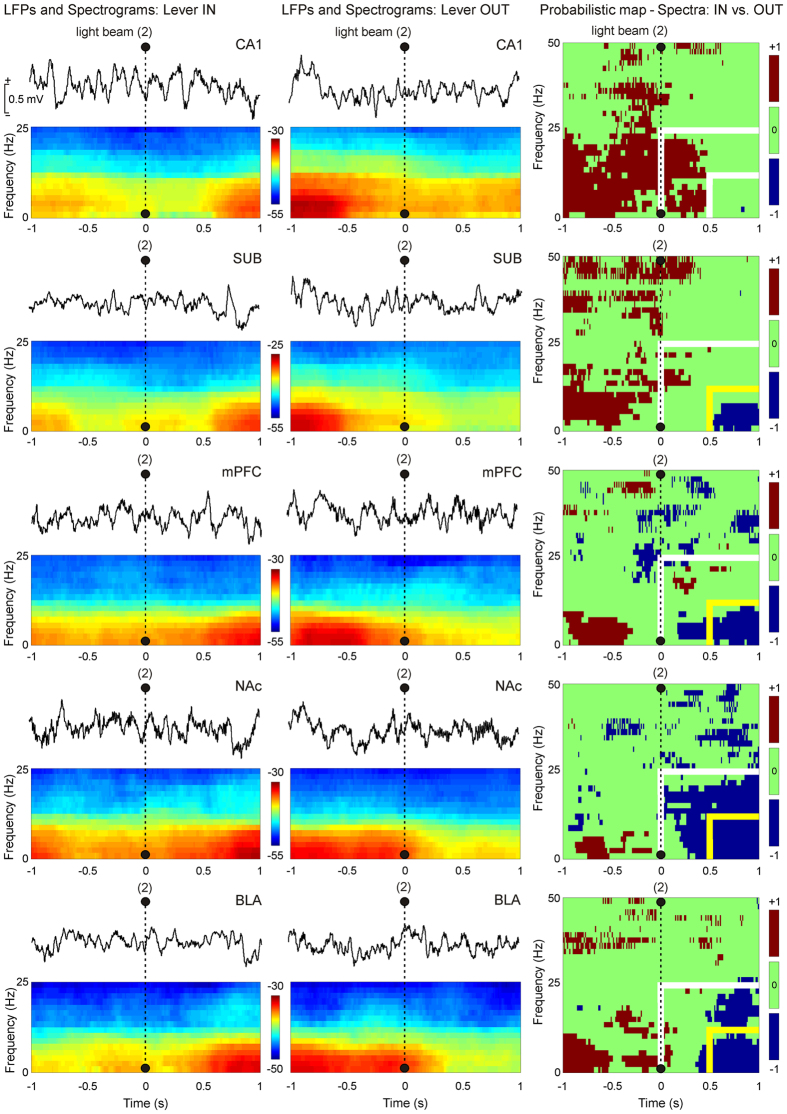
Dynamic changes in LFP activities at the five recording areas for the two different experimental conditions (Lever IN or Lever OUT). Mean LFP recordings and time-frequency representations (spectrograms; *N*_*T*_ × *K* = 300 tapered Fourier transforms) corresponding to the five recording areas are illustrated in the left (Type A trials, Lever IN) and middle (Type B trials, Lever OUT) panels. Note that the maximum values of spectral power (see the color bar) appeared at the end (for Lever IN) or the beginning (for Lever OUT) of the LFP time window [from −1 s before (−1 s) to 1 s after (+1 s) the light beam (2) was cut]. Whereas spectral powers of LFPs during type A trials (Lever IN) increased, the spectral powers of LFPs during type B trials (Lever OUT) decreased. In addition, the fundamental contribution to the spectral power is determined by delta (0.1–3 Hz) and theta (3–12 Hz) bands. In the right panels are represented the probabilistic maps for the multiple comparisons between pairs of spectrograms (Lever IN vs. Lever OUT). The colors blue [−1; estimate of spectral power for Lever OUT (*E*_OUT_) ≪ estimate of spectral power for Lever IN (*E*_IN_)] and brown (+1; *E*_OUT_ ≫ *E*_IN_) denote significant statistical differences (*P* < 0.05, accept H0 of different population spectra), and green (0; *E*_OUT_ ≈ *E*_IN_) indicates no significant differences (*P* > 0.05, reject H0 of different population spectra). Note that the probability density of inferences of type −1 (accept H0, *E*_OUT_ ≪ *E*_IN_, blue color) is predominant in the 2D range [0.5–1 s; 0–25 Hz] for all the brain sites (except at CA1), which means that the spectral power of the LFP recordings decreased significantly (jackknifed estimates of the variance, *P* < 0.05) during type B trials (Lever OUT).

For a more-precise dynamic analysis of spectral powers computed from LFPs, moving time windows of 500 ms (shifted in increments of 10 ms) were selected and then multi-tapered Fourier transforms (see Methods) were calculated. Figure 6 illustrates mean LFPs recorded from the five selected cortical (CA1, SUB, and mPFC) and subcortical (NAc and BLA) sites during Lever IN and Lever OUT situations from 1 s before to 1 s after the 2nd light beam was crossed. The illustrated spectrograms correspond to 300 tapered Fourier transforms, each corresponding to the average of 60 trials × 5 tapers (see Methods). Collected results indicate that the maximum power values appeared at the end (for Lever IN) or the beginning (for Lever OUT) of the selected time window. In addition, spectral powers of the LFPs during type A trials (Lever IN) increased (i.e., when the animal was closer to the lever site) in the delta (0.1–3 Hz) and theta (3–12 Hz) bands. In contrast, the spectral powers of the LFPs during type B trials (Lever OUT) decreased for these two bands.

**Figure 7:**
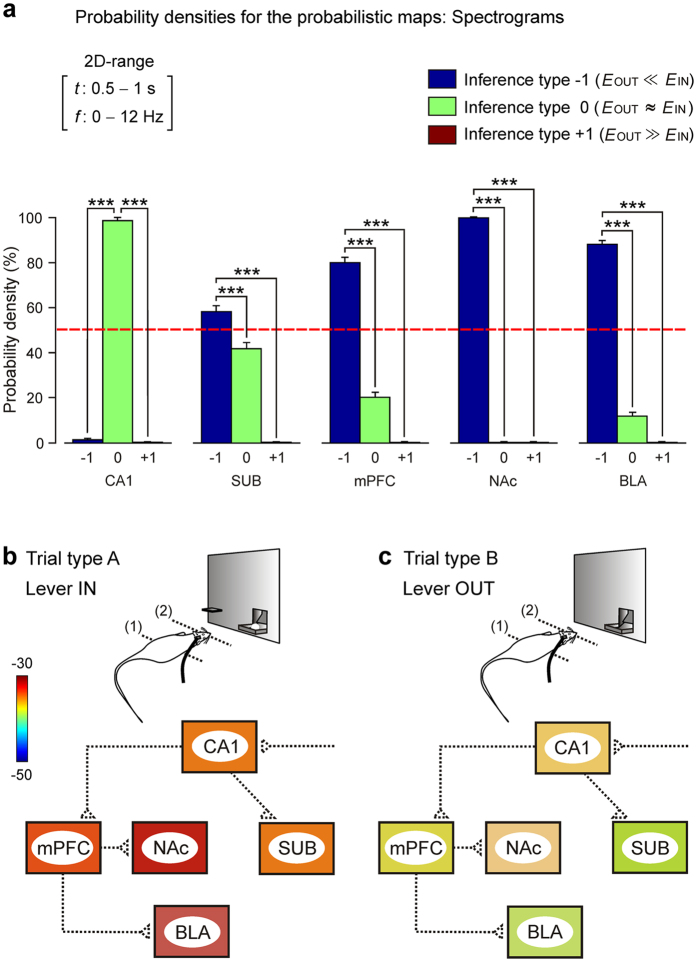
Probability density histograms for Lever IN vs. Lever OUT conditions. (**a**) Probability densities (in %) for the probabilistic maps corresponding to the five spectrograms illustrated in Fig. 6. Note that, in the 2D range [0.5–1 s; 0–12 Hz], the probability density of inferences of type −1 (blue bar in the histogram, accept H0, *E*_OUT_ ≪ *E*_IN_) is predominant (probability density >50%, *P* < 0.001) for all the brain sites (except at CA1), which means that the spectral power of LFP recordings decreases significantly (jackknifed estimates of the variance, *P* < 0.05) during type B trials (Lever OUT). For each histogram the same optimal 2D range (time-frequency) is reported. Data are represented by the Mean ± SEM and the significance level is indicated (***P < 0.001). Dashed red line indicates the 50% of probability density. (**b,c**) The left diagram (**b**) summarizes changes in spectral power that took place for situation A (lever always in), whilst the right diagram (**c**) illustrates changes in power that took place for situation B (lever always out) in an unpredictable situation. Averaged values of the spectral power (see the color bar) in delta and theta bands during the 0.5–1 s period after crossing the 2nd light beam as illustrated in Fig. 6.

In Fig. 6 (right panels) are also illustrated differences in power spectra (1–50 Hz) for LFP recorded from 1 s before to 1 s after the 2nd light beam was crossed. The high frequency gamma band (50–100 Hz) was excluded from this analysis because of the absence of any significant change during the two experimental situations illustrated in Fig. 5b,c. In general, the probabilistic maps indicate a significant decrease in the spectral power of the LFP recordings during type B trials (Lever OUT) for all recording sites in the delta and theta bands, apart from the hippocampal CA1, mostly during the last 0.5 s of the analyzed period (see the small yellow-marked squares in Fig. 6, right set of probabilistic maps, and Fig. 7a). In Fig. 7a is illustrated the distribution of probability densities corresponding to the probabilistic maps shown in Fig. 6 (right panels). Note that the probability density of inferences of type −1 [blue bar, accept H0, *E*_OUT_ (estimate of spectral power during Lever OUT condition) ≪ *E*_IN_ (estimate of spectral power during Lever IN condition)] is predominant (probability density > 50%, *P* < 0.001) in the 2D range [0.5–1 s; 0–12 Hz] for all the brain sites (except at CA1), which means that the spectral power of LFP recorded at SUB, mPFC, NAc, and BLA decreases significantly (jackknifed estimates of the variance, *P* < 0.05) during the Lever OUT condition.

In summary, the close vicinity of experimental animals to the site at which the lever was expected evoked a significant decrease in power (for delta and theta bands) and non-significant changes in gamma (25–100 Hz) bands from LFPs at most (SUB, mPFC, NAc, and BLA) of the recording sites (Fig. 7b,c).

## Discussion

A feature of adaptive behaviors necessary to select new action plans in an already acquired learned situation is the early detection of unpredictable cues appearing in the environment^1^,^2^,^47^. To understand cognitive processes by which the brain performs the comparison between already learned and new cues or related contexts, it seems necessary to study the ongoing activities in brain neuronal ensembles (and in their corresponding synaptic contacts) involved in these processes. Cortical structures such as CA1, SUB, and mPFC, as well as their afferent and efferent connecting networks, have been reported to participate in novelty detection and/or in the generation of newly adapted behaviors^48^,^49^,^50^. Our data clearly show that these structures and their corresponding synaptic contacts participate in the retrieval and/or performance of already learned behaviors (e.g., the Lever IN situation) and are also involved in the detection of cue changes during the learning task and their consequent behavioral adaptation (e.g., the Lever OUT situation). Similar changes in strength has been found in CA1, SUB and mPFC areas of rats presented with the same unpredictable situation^51^. The accurate analysis for the different frequency bands in the probabilistic maps for the multiple comparisons between pairs of spectrograms developed in the present study allowed to reach more detailed conclusions than in this previous study.

The hippocampal CA1 receives predictions from previous experiences through Schaffer collaterals^24^,^52^. In addition, this region is specifically related to the maintenance and retrieval of familial spatial contexts^53^,^54^ and it has been proposed that CA1 computed novelty through a comparison between stored experiences and new sensory inputs^7^. Perforant pathway inputs to CA1 (PP-CA1 synapse) seem to code sensory information arriving from many other cortical regions^55^,^56^. The present results partly support these previous contentions, because we detected an increase in fPSP slopes evoked at the PP-CA1 synapse related to different spatial contexts (i.e., Skinner vs. baseline boxes). Although the disruption of PP-CA1 synapses impairs the presence of novelty components in spatial memories^57^—a phenomenon related to gamma frequencies^58^— sensory inputs received in the Skinner box did not evoke significant differences in PP-CA1 fPSP slopes between control, Lever IN, and Lever OUT situations. Our data show that the power of CA1 delta and low-theta bands was higher when the rat pressed the lever in the IN situation, related to the performance of precise motor activities^59^. Finally, CA1 recording electrodes presented higher spectral power in the high-gamma band related to the decision of lever pressing^60^ compared with the Lever OUT trials.

The final relay in the synaptic loop between the entorhinal cortex and the dorsal hippocampus is the SUB, which is principally involved in information processing of space, movement, and related memories. Indeed, the firing of subicular cells correlates best with object locations in the nearby environment^61^. In agreement with that, we have detected a significant increase in fPSP slopes in the CA1-SUB synapse during animals’ approaches to the lever site (even if the lever was OUT) as compared with slopes collected for control situations. Nevertheless, significant differences in CA1-SUB fPSPs were observed for Lever IN and Lever OUT conditions. In fact, Potvin *et al.*^37^ have shown that the dorsal SUB is necessary for the proper functioning of spatial location memories, but they did not find significant differences in novelty detection with objects or odors in an open field task during 3-minute recordings, an experimental design rather difficult to compare with the present experiments^37^. Furthermore, in common with other authors^62^, we detected a decrease in the power of delta and theta bands in LFPs recorded in hippocampal SUB during the unpredictable task, but, in contrast to recordings carried out in CA1, no increase in power of the gamma band.

The dorsal hippocampus and the mPFC seem to process spatial memories in parallel^49^. In addition, one of the main roles of mPFC is the organization and processing of timed motor and emotional behaviors^28^,^49^,^63^,^64^. Given the functional heterogeneity of the prefrontal cortex^65^,^66^ it is important to point out that implanted mPFC electrodes were located in the prelimbic area. It has been already reported that the hippocampus (CA1, SUB) innervates the dorsal prelimbic area^67^. Additional anatomical evidences suggest that, in the rat, hippocampal CA1 afferents innervates both excitatory (projecting) pyramidal neurons and inhibitory interneurons^68^. According to the present results, the strength of CA1-mPFC synapses was maximum before the decision of pressing the lever and decreased when the lever disappeared and the animal was unable to finish the projected task. This was correlated with a power decrease in delta, theta, and beta frequencies during the unpredictable situation. Several authors^15^,^19^,^69^ have reported similar results in a task in which the animals had to learn an object-in-place rule or a maze-based task. They reported specifically that after the task had been learned, CA1 and mPFC neurons reached maximum firing synchronization in the theta band before the decision-making point.

mPFC, NAc, and BLA areas seem to be coordinated during memory consolidation involving motivational aspects^70^. NAc neurons encode motivational processes such as instrumental learning^71^,^72^ as well as reward predictive cues and food reward delivery^73^. Electrical stimulation or dopamine infusion in NAc increases neuronal firing and is related to an increase in attention to relevant environment cues^27^,^74^,^75^. The observed increase in fPSP slopes in the mPFC-NAc synapse during Lever IN and control situations supports the reported increase in attention to perform the learned behavior in a specific environment. In accordance, disturbing the process during the Lever OUT situation decreased fPSP slopes in this synapse. As an effect of novelty, the power spectrum of LFPs recorded in the NAc decreased dramatically for delta, theta, and beta bands during lever OUT. Interestingly, novelty detection in humans evokes a decrease in theta and beta bands in the NAc^76^.

Previously reported results suggest that BLA is a critical structure for instrumental conditioning, mediating the encoding of sensory aspects of particular motivational outcomes^77^,^78^,^79^. The increase in strength in the mPFC-BLA synapse during the performance of already learned behaviors and its subsequent decrease in the unpredictable task support the proposal of the specific involvement of the BLA (and its connections with the mPFC) in the mechanism of representation of action-outcome. Specific lesions of the BLA had no effect on the acquisition of lever pressing but attenuated the impact of reward devaluation^77^. Likhtik *et al.*^80^ have suggested that selective tuning of mPFC and BLA neural firing provides a safety-signaling for learned fear and innate anxiety situations^80^. Similarly, and with regard to mPFC and NAc relationships, we detected a decrease in spectral power in delta, theta, and beta bands when the animal was close to the point at which the lever was expected to be for the OUT situation, as compared with power computed during the Lever IN trials.

On a whole, our data suggest that unpredictable situations like that presented here to operant-trained rats are significantly reflected in the synaptic outputs from CA1 to SUB and mPFC, as well as the projections of the latter to NAc and BLA. In opposition, the PP-CA1 synapse seemed to be mostly related to contextual differences between baseline and Skinner-box situations (see Fig. 3b–e). In addition, an increasingly suggested hypothesis is that neural information is coded —and transmitted between different cortical and subcortical areas— using LFP oscillations^81^ and that neurons may exhibit different preferred oscillatory activities for different behavioral situations^82^. With regard to these suggestions, we have shown here that there are statistically different oscillations in the five structures included in the study to form a cognitive functional state corresponding to the unpredicted situation. Indeed, collected results indicate that during the already learned situation, SUB, mPFC, NAc, and BLA presented larger spectral powers than during the unpredictable availability of a reward-related cue, while CA1 did not show significant changes. The reported fast changes in synaptic strength and in the oscillatory properties of the recording sites suggest a prompt preparation of involved neural circuits to the generation of adapted behaviors to the new environmental constrains (see Figs 3b–e and 7b,c).

## Methods

### Experimental subjects

Experiments were carried out with male Wistar rats (3 months old, 250–300 g) obtained from an official supplier (University of Granada Animal House, Granada, Spain). Before surgery, animals were housed in separate cages (n = 4 per cage). Rats were kept on a 12:12 h light-dark cycle with constant ambient temperature (21 ± 1 °C) and humidity (50 ± 7%). Unless otherwise indicated, food and water were available *ad libitum*. Electrophysiological and behavioral studies were carried out in accordance with the guidelines of the European Union Council (2010/63/EU) and Spanish regulations (BOE 34/11370-421, 2013) for the use of laboratory animals in chronic experiments. Experiments were also approved by the local Ethics Committee (01/2011) of the Pablo de Olavide University (Seville, Spain).

### Surgery

Rats were divided in four groups (n = 10 animals/group) and implanted with: (i) bipolar stimulating electrodes in the left PP and recording electrodes in the ipsilateral hippocampal CA1 area; (ii) stimulating electrodes in the left CA1 area and recording electrodes in the ipsilateral mPFC and SUB; (iii) stimulating electrodes in the left mPFC and recording electrodes in the ipsilateral NAc and the BLA; and (iv) recording electrodes in the left CA1, SUB, mPFC, NAc, and BLA areas (Fig. 1a–c).

Before surgery, rats were fasted overnight. For surgery, animals were anesthetized with 0.8–1.5% isofluorane delivered from a calibrated Fluotec 5 (Fluotec-Ohmeda, Tewksbury, MA, USA) vaporizer at a flow rate of 1–2 L/min following a protective injection of atropine sulfate (0.1 mg/0.1 kg, i.m.). As illustrated in Fig. 1a–c, animals were implanted with stimulating (bipolar) and recording (tetrodes) home-made electrodes in the following left brain sites^83^: (i) PP, dorsomedial aspect of the left angular bundle (6.8 mm posterior, 3 mm lateral and depth of 2 mm) (ii) dorsal CA1 area (4.6 mm posterior, 2.2 mm lateral and depth of 2.3 mm); (iii) dorsal SUB (6.8 mm posterior, 4 mm lateral and depth of 2.5 mm); (iv) prelimbic mPFC (3.2 mm anterior, 1 mm lateral, and depth of 3.8 mm); (v) NAc (1.7 mm anterior, 1.5 mm lateral, and depth of 6.5 mm); and (vi) BLA (2.8 mm posterior, 4.8 mm lateral, and depth of 7.6 mm). For all structures, anterior, posterior and lateral stereotaxic references were taken from bregma and depth was measured from brain surface.

Electrodes were made from 25 μm, Teflon-coated tungsten wire (Advent Research Materials, Eynsham, UK). The recording electrodes (≥8 per animal) were fixed at the site where a reliable fPSP was recorded. As a control to detect unwanted contaminations of LFPs by electrical muscle activities, animals were also implanted with bipolar electromyography (EMG) recording electrodes in the left whisker muscles. These electrodes were made from 50 μm, Teflon-coated, annealed stainless steel wire (A-M Systems, Carlsborg, WA, USA), with the tips bared of the isolating cover for ≈0.5 mm. The electrode tips were bent as a hook to facilitate a stable insertion in the implanted muscles. Finally, animals were implanted with a 0.1 mm bare silver wire affixed to the parietal bone as ground. All wires were soldered to three four-pin sockets (RS Amidata, Madrid, Spain), and the sockets were fixed to the skull with the help of three small screws and dental cement^24^,^84^.

### Recording and stimulation procedures

LFP recordings were carried out using Grass P511 differential amplifiers with a bandwidth of 0.1 Hz–10 kHz (Grass-Telefactor, West Warwick, RI, USA). fPSPs were evoked in the different recording sites (CA1, SUB, mPFC, NAc, and BLA) by single 100 μs, square, biphasic (negative-positive) pulses applied to the selected stimulation sites (PP, CA1, or mPFC). Stimulus intensities ranged from 50 μA to 350 μA. For each animal, the stimulus intensity was set at 30–40% of the intensity necessary for evoking a maximum fPSP response. During the recording sessions, stimuli were presented with a minimum interval of 20 s.

### Operant conditioning of implanted rats

Training and testing took place in modified Skinner box modules (n = 3) measuring 29.2 cm × 24.1 cm × 21 cm (MED Associates, St. Albans, VT, USA). Each Skinner box was provided with a division wall (Fig. 1d) located between the lever and the feeder modules and with two light beams placed at 10 cm (1) and 2 cm (2) from the lever (Fig. 2a). Each operant box was housed within a sound-attenuating chamber (90 cm × 55 cm × 60 cm), which was constantly illuminated (19 W lamp) and exposed to a 45 dB white noise (Cibertec, S.A., Madrid, Spain). Skinner boxes were equipped with food dispensers from which pellets (MLabRodent Tablet, 45 mg; Test Diet, Richmond, IN, USA) could be delivered by pressing a lever.

Before training, rats were handled daily for 7 days and food-deprived to 80–85% of their free feeding weight. Once the desired weight was reached, animals were placed in the Skinner box for 20 min and allowed to press the lever to receive pellets from the food tray using a fixed-ratio (FR 1:1) schedule (Fig. 1e). The start and end of each session was indicated by a tone (2 kHz, 200 ms, 70 dB) provided by the loudspeaker located in the recording chamber. The selected criterion was to press the lever >100 times/session for two consecutive sessions (Fig. 1f). Animals were allowed a maximum of five days to reach criterion. Conditioning programs, lever presses, and delivered reinforces were controlled and recorded by a computer, using a MED-PC program (MED Associates).

Animals were trained until reaching the selected criterion with the wire system connected to the implanted sockets, but recordings carried out in these sessions will not be considered in the present study.

### Experimental design of the unpredictable task

Once the animal had reached the selected criterion and maintained it for ≥3 training sessions, it was switched to the unpredictable task, for a single 21-minute session. fPSPs were evoked and recorded in four different consecutive situations (Fig. 2a,b): (i) *I. Baseline*: the animal was placed for 3 min in an auxiliary Perspex box (36 cm × 25 cm × 20 cm) located beside the Skinner box used for the experiments; the auxiliary box presented empty walls; (ii) *II. Control*: stimuli were presented when the animal was resting outside the lever and feeder areas inside the Skinner box; (iii) *III. Trial type A, Lever IN*: the lever was available when the animal approached it across the strip created with the two (1, 2) light beams; stimuli were presented when the animal cut the 2nd light beam (black dots); and (iv) *Trial type B, Lever OUT*: the lever was removed when the animal’s head cut the 1st light beam; also in this case, electrical stimulation of the selected synapses was carried out when the animal’s head crossed the 2nd light beam (black dots). In the four situations, electrical stimulation was presented with intervals >20 s.

The single session with trials in which the availability to the reward-related cue was unpredictable included 3-min periods across the session and lasted for a total of 21 min (Fig. 2b). In the odd blocks (1, 3, 5 and 7), the lever was always available for situation Type A trials, whilst in the even blocks (2, 4 and 6), it was available or removed at random for situation Type A or Type B trials. Inside each block, all the trials were the same (either type A or type B).

Each animal received just one unpredictable session in which either it was stimulated at the implanted site and the evoked fPSPs were recorded during the above-mentioned four situations (baseline, control, and trials type A and B) or LFPs were recorded simultaneously from all implanted sites during trials type A and B (see Fig. 1b).

### Histology

At the end of the experimental sessions, rats were deeply reanesthetized (sodium pentobarbital, 50 mg/kg) and perfused transcardially with saline and 4% phosphate-buffered paraformaldehyde. Their brains were removed, postfixed overnight at 4 °C, and cryoprotected in 30% sucrose in PBS. Sections were obtained in a microtome (Leica) at 50 μm. Selected sections including the implanted (stimulating and/or recording) sites were mounted on gelatinized glass slides and stained using the Nissl technique with 0.1% toluidine blue to determine the location of stimulating and recording electrodes (Fig. 1c).

### Data collection, analysis, representations, and statistical tests

fPSPs, LFPs, and 1-volt rectangular pulses corresponding to lever presses, pellet delivery, lever in/out situations, and brain stimulations were stored digitally on a computer through an analog/digital converter (CED 1401 Plus; Cambridge Electronic Design, Cambridge, UK). Data were analyzed off-line for quantification of fPSPs, LFPs, and the animals’ performance in the Skinner box (Fig. 1d,e), using the commercial computer programs Spike 2 and SIGAVG (from Cambridge Electronic Design) and the video capture system.

Only data from successful animals (i.e., those that allowed a complete study with a proper functioning of both recording and stimulating systems) were computed and analyzed. Following previous descriptions^85^, the slope of evoked fPSPs was calculated as the ratio between the difference of amplitudes (in mV) and the corresponding difference of times (in ms) for the selected points from fPSP recordings (i.e., mV/ms). Five successive fPSPs were averaged, and the mean value of the slope during the rise-time period (i.e., the period of the slope between the initial 10% and the final 10% of the fPSP) was determined (Figs 2c and 3a).

LFP epochs each lasting 2 s [1 s before (−1 s) and 1 s after (1 s) the cutting of the light beam (2), see Fig. 4a,b] were selected during the task performance in the Skinner box for both (A: Lever IN, B: Lever OUT) types of trial. This precise timing was selected from preliminary experiments indicating that the rat reached the lever at the end of this selected LFP epoch. In particular, two intervals from these 2-second selected epochs were further analyzed: (i) the 2nd half of the 2 s (0–1 s); and (ii) the last 500 ms of the 2 s (0.5–1 s). Analysis in the frequency domain was carried out for the following frequency bands: delta, 0.1–3 Hz; theta, 3–12 Hz [divided in two sub-bands of 3–8 Hz (low-theta) and 8–12 Hz (high-theta)]; beta, 12–25 Hz; and gamma, 25–100 Hz [divided in two sub-bands of 25–50 Hz (low-gamma) and 50–100 Hz (high-gamma)]^34^. The algorithm included the analysis of mean values of the spectral powers between the different frequency bands of each LFP epoch (e.g., approximately 2.0 s, 1.0 s, or 0.5 s before the lever was reached) and the analysis of mean values of the spectral powers for the same frequency band between the different experimental conditions (e.g., between type A: Lever IN and type B: Lever OUT trials).

All computational tools for neurophysiological signals processing in the frequency domain (Figs 4c and 5b,c) and the analytical approach of multi-taper Fourier transforms (Figs 6 and 7) in the time-frequency domain were designed and developed by us using home-made programs^34^,^51^,^86^,^87^ written in MATLAB platform (version 8.3, R2014a. The MathWorks, Natick, MA, USA) and customized scripts of Chronux^88^,^89^,^90^ software (version 2.11, R2014. Website: http://chronux.org/), respectively. Recent spectral applications that include the raster displays^34^ of the power spectra (see Fig. 4c) and the probabilistic maps^51^ for the comparison between pairs of spectrograms (see Fig. 6) were also incorporated to this work. The time-frequency analyses of LFP recordings and the multiple comparisons of spectra of LFPs recorded from the different brain sites (CA1, SUB, mPFC, NAc, and BLA) and experimental conditions (Lever IN and Lever OUT trials) were performed taking into account *N*_T_ = 60 trials and *K* = 5 tapers for each averaged spectrogram (Fig. 6). Spectrograms were computed using a moving time window (*T*) of length 500 ms (shifted in 10-ms increments) and 6 Hz of frequency bandwidth (*W*), allowing a time-bandwidth product of 3. These parameters verify the number of selected tapers (i.e., *K* = 2 × *T* × *W* − 1 tapers or windowing functions) and the time-frequency resolution for the spectral representations (Fig. 6; time, *dt* = 10 ms; frequency, *df* = 1.75 Hz).

The multi-taper estimates of the spectrum with *N*_T_ trials and *K* tapers were based on computing *N*_*T*_ × *K* Fourier transforms that determined an appropriate number of degrees of freedom *dof* = 2 × *N*_*T*_ × *K* for all the computations. Since the trials can be assumed to be interchangeable, the statistical estimates can be obtained by leaving out one taper of one trial in turn. This procedure has been shown to be useful in the analysis of a wide variety of time series, including neurophysiological recordings^91^,^92^,^93^,^94^,^95^.

Because the number of trials affects spectrum estimations, these magnitudes were transformed using the variance stabilization method^88^ to reduce bias in statistical estimations of the *Z*-statistic during the multiple comparisons. The transformed spectrum is given by the following equation (Eq. 1):


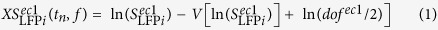


where 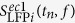 is the auto-spectra of LFP*i* (recorded from each selected site, see Fig. 6). Similar equations hold for 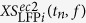 in the second experimental condition (*ec*2, for example, the Lever OUT condition). The algorithm proposed here includes a redefinition of the tapered Fourier transforms (dimension: time × frequencies × number of samples) for *t*_*n*_ discrete values of the spectrogram time window. Thus, the proposed *Z*-statistics (Eq. 2) were explicit (*t*_*n*_, *f*) -dependent functions of the time and frequency:


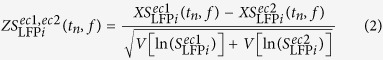


These computations were used to generate the probabilistic maps (see Eq. 2 and the right panels in Fig. 6) for the dynamic analysis of the levels of significance (*P*) during the multiple comparisons of the spectrograms in two different experimental conditions (Lever IN vs. Lever OUT). Note that these probabilistic maps have the same time-frequency resolution as spectrograms (left and middle panels in Fig. 6). In the color scales to the right of the maps, the colors blue [inference of type −1; *E*_OUT_ (estimate of spectral power during Lever OUT condition) ≪ *E*_IN_ (estimate of spectral power during Lever IN condition)] and brown (inference of type +1; *E*_OUT_ ≫ *E*_IN_) denote significant statistical differences (*P* < 0.05), while green (inferences of type 0; *E*_OUT_ ≈ *E*_IN_) indicates no significant differences (*P* > 0.05). Note also that the above criterion for the probabilistic maps during the comparison of two experimental conditions (e.g., *ec*1 for Lever IN; *ec*2 for Lever OUT) assesses the null hypothesis (H0)—i.e., 1 for accept H0 (*P* < 0.05) of different population spectra (Eq. 3), and 0 for reject H0 (*P* ≥ 0.05).


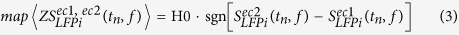


Computed results were processed for statistical analysis using the customized mini-packages of Chronux^89^,^90^ for the jackknifed estimates of the variance and of Z-statistics (Figs 6 and 7). In addition, the ‘Multivariate Statistics’ MATLAB Toolbox and the Sigma Plot 11.0 package (Sigma Plot, San Jose, CA, USA) for Windows were used.

For multivariate statistics assessments both parametric [ANOVA *F*-tests, with or without repeated measures (RM); e.g., see Figs 5b,c and 7a] and non-parametric [ANOVA tests on Ranks, with RM (Friedman ANOVA) or without RM (Kruskal-Wallis ANOVA); see Fig. 1f] methods were used to assess the statistical significance of differences between groups, followed by the appropriate test (Holm-Sidak, or Tukey, or Student-Newman-Keuls, in this order of priority) for all the pair wise multiple-comparison analyses^34^,^96^.

In general, when the normality (Shapiro-Wilk, or Kolmogorov-Smirnov test) and equal variance of the errors (Levene Median test) assumptions were satisfied, the significance (*P*-value) and the statistic *F*_[(*m*−1),(*m*−1)×(*n*−1),(*l*−*m*)]_, with its corresponding orders *m* (number of behaviors), *n* (number of animals), and *l* (number of multivariate observations) were reported. In addition to the values of *F* and *P*, the “effect sizes” were informed depending on the partial η^2^ index for One-way RM ANOVA test with multiple groups (Fig. 3b,c), and according to η^2^ and Cohen’s d indices^97^ for One-way ANOVA without RM between two groups (Figs 5b,c and 7a). The reports of Cohen’s d value included the 95% of the confidence interval (CI). Use of an effect size with a CI conveys the same information as a standard test of statistical significance, but with the emphasis on the significance of the effect, rather than the sample size. If this CI includes zero, then that is the same as saying that the results is not statistically significant. If, on the other hand, zero is outside the CI range, then it is statistically significant.

When the normality assumption was not verified, the significance (*P*-value) of the *Chi-square* statistic was calculated using the ranks of the data rather than their numeric values^98^. In addition, *Chi-square* statistic and the sample size of data were used to estimate the corresponding effect size index. Finally, the parametric (see Fig. 3b–e) and non-parametric (see Fig. 1f) methods were also applied to the data considering the Skinner sessions as repeated measures.

Unless otherwise indicated, data are represented by the Mean ± SEM. For all the statistical tests, the significance level (*P*-value) was indicated. It is common to declare a result significant if the estimated *P* value is <0.05 (*), 0.01 (**), or 0.001 (***).

## Additional Information

**How to cite this article**: Fernández-Lamo, I. *et al.* Functional states of rat cortical circuits during the unpredictable availability of a reward-related cue. *Sci. Rep.* **6**, 37650; doi: 10.1038/srep37650 (2016).

**Publisher’s note**: Springer Nature remains neutral with regard to jurisdictional claims in published maps and institutional affiliations.
